# Oxidative Stress in the Reproduction of Mammals

**DOI:** 10.3390/antiox14030306

**Published:** 2025-03-02

**Authors:** Paul Juan Jacobs, Nigel Charles Bennett

**Affiliations:** Department of Zoology and Entomology, Mammal Research Institute, University of Pretoria, Pretoria 0002, South Africa

## 1. Introduction

Reproduction is a fundamental biological process, but it is also vulnerable to oxidative stress, which is a consequence of an imbalance between reactive oxygen species and antioxidant defences [1,2]. Oxidative stress arises when cells generate ROS and, despite being relevant for cell signalling and immune function, excessive levels can damage lipids, proteins, and DNA [3]. In terms of reproduction, oxidative stress processes can intensify through gamete production, pregnancy, or parental care, but can also show contrasting patterns of reduced oxidative stress [4,5]. This imbalance can ultimately affect reproductive health, where elevated oxidative stress in both males and females has been linked to reduced sperm and egg quality, impaired embryo development, pregnancy complications, as well as lower offspring survival [1,2]. The consequences of oxidative stress highlight the importance of maintaining oxidative balance for successful reproduction and health.

This raises important research opportunities. Firstly, monitoring and ameliorating oxidative stress through antioxidant supplementation during reproduction can provide insights into how reproductive health can be maintained or enhanced. Secondly, exploring life history trade-offs can reveal how organisms allocate resources between reproduction and longevity. Finally, and not least, examining the consequences of oxidative stress antagonists on reproductive status is essential to determine whether interventions improve fertility or inadvertently disrupt natural reproductive processes. This Special Issue features research covering these areas that explores how oxidative stress influences reproductive processes in mammals.

## 2. Overview of Published Articles

Reducing oxidative stress, such as through antioxidant supplementation during reproduction, can provide insights into how reproductive health can be maintained or enhanced [1,2]. Alexandru et al. [Contribution 1] highlighted the role of vitamins and antioxidants in enhancing fertility and fertility preservation of women diagnosed with gynaecological cancers. They found that vitamin A supports oocyte integrity, whereas vitamins C and E reduce oxidative stress in ovarian tissues, and vitamin D3 improves ovarian reserves and modulates inflammation. Additionally, coenzyme Q10 boosts mitochondrial function, reducing DNA damage, and increasing oocyte viability and fertilization potential. Cai et al. [Contribution 2] found that the supplementation of β-nicotinamide mononucleotide (NMN) enhanced NAD+ and ATP levels in granulosa cells and protecting them from lipopolysaccharide-induced apoptosis, oxidative stress, and mitochondrial dysfunction while restoring ovarian steroidogenesis through the AMPK/mTOR pathway. This suggests NMN plays a key role in regulating dominant follicle selection and reproductive function by mitigating inflammatory and metabolic stress, protecting granulosa cells. Chavas et al. [Contribution 3] demonstrated that bovine spermatozoa exposed to oxidative stress may benefit from the supplementation of pterostilbene in a dose-independent manner, which reduces lipid peroxidation and increases intracellular glutathione levels. This supplementation also improved energy production by restoring ATP and AMP/ATP levels and promoted autophagy through AMPK activation. These effects collectively inhibited apoptotic cell death, highlighting pterostilbene’s protective role on bovine spermatozoa during freezing and thawing processes. Li et al. [Contribution 4] supplemented sow diets with VD_3_ and 25-OH-D_3_. This improved plasma oxidative stress status, enhanced placental antioxidant capacity and nutrient transport, and also reduced placental inflammation, where the effects of 25-OH-D_3_ were most pronounced in their protective properties.

The early monitoring and detection of oxidative stress enables the better management of related conditions and provides insights into improving health outcomes through targeted interventions. The study by Kohzadi, Kubow, and Koski [Contribution 5] investigated the relationships between foetal growth and oxidative stress, amniotic fluid antioxidant capacity, and minerals, with a focus on prenatal supplementation, and was monitored using ultrasound. The findings revealed that certain minerals in the amniotic fluid were significantly correlated with foetal growth parameters such as foetal weight and femur length. The findings suggest that oxidative stress plays a significant role in foetal development and that monitoring amniotic fluid components may be an important component in the assessment of foetal health in a non-invasive manner.

Exposure to polycyclic aromatic hydrocarbons may result in reproductive infertility, which can come from natural sources and from the work environment [6]. The work by Peña-García et al. [Contribution 6] investigated how exposure to polycyclic aromatic hydrocarbons can affect the reproductive health of male workers who operate in solar thermal plants. The findings showed that a significantly higher seminal leukocyte infiltration occurred, but a lower activity in seminal plasma of superoxide dismutase (SOD) and a reduced glutathione/oxidised glutathione (GSH/GSSG) ratio arose in these individuals. However, despite the disruption of the oxidative balance, the sperm cellularity of the workers, either quantitatively (sperm count) or qualitatively (motility, vitality, morphology, or cellular DNA fragmentation) was surprisingly unaffected. This study provides evidence that hydrocarbons in the work environment can disrupt oxidative stress, but that overall reproductive function remains intact.

Life history trade-offs are a key research area in the reproductive trade-off of animals as resources are finite. Oxidative stress has been proposed as a proximate mechanism that mediates these life history trade-offs. The work by Birch et al. [Contribution 7] provides further evidence for the oxidative shielding hypothesis, which posits that the diminishment of oxidative damage by breeders may act as an adaptive, pre-emptive measure to protect the next generation from oxidative insults [5]. Among male banded mongooses (*Mungos mungo)*, breeding males exhibited lower ejaculate MDA levels, supporting the oxidative shielding hypothesis and suggesting enhanced antioxidant protection during reproductive activity. Additionally, an increase in vitamin E levels close to mate competition indicates a potential boost in somatic antioxidant defence, further reinforcing the idea of strategic oxidative stress management. These findings highlight the possibility that oxidative shielding plays a crucial role in male reproductive success, with potential contributions from both sexes yet to be explored. The work by Jacobs et al. [Contribution 8] further investigated potential differences in the life history trade-offs of alternative reproductive tactics in male Cape Ground squirrels (*Xerus inauris*) through oxidative stress. Male squirrels employ two distinct reproductive tactics or alternate reproductive tactics: band males, who join same-sex roving groups in search of oestrous females [7], and natal males who, at maturity, delay dispersal and remain with their natal group and provide alloparental care [8,9]. They found that male reproductive strategies influenced oxidative stress profiles due to differences in metabolic rate, activity, and foraging behaviour, with roaming males experiencing higher oxidative demands than natal males. Regardless of the strategy employed, oxidative stress declined with age, potentially due to survival-based selection pressures favouring individuals with better oxidative balance. These findings highlight the role of metabolic demands and life-history strategies in shaping oxidative stress dynamics across different male reproductive tactics.

## 3. Conclusions

Oxidative stress plays a vital role in the health and well-being of mammals, particularly during reproduction. Previous research on antioxidant supplementation has demonstrated its ability to alleviate oxidative stress, significantly benefiting reproducing mammals. Additionally, identifying reliable methods to assess oxidative stress is crucial for enhancing these benefits. This Special Issue expands on the body of knowledge on the advantages of supplementation in reproductive health by exploring novel, non-invasive techniques for the detection of oxidative stress during reproduction.

Less understood, however, is the reproductive impact of hydrocarbons on mammalian fertility. Findings from this Special Issue suggest that, despite prolonged exposure to environmental hydrocarbons, known to induce oxidative stress, and although oxidative stress was induced, fertility was not compromised. Gaining deeper insights into how oxidative stress-inducing environmental factors affect reproductive health, and how oxidative stress levels change following exposure, can contribute to the creation of healthier working environments.

The complex, non-linear relationship between oxidative stress and reproduction in wild mammals makes this an intriguing area of research. This Special Issue provides further evidence supporting this non-linear dynamic, revealing that the older individuals and males of Cape Ground squirrels with alternative reproductive strategies exhibit lower oxidative stress, in contrast to the expected pattern of increased oxidative stress with age. In banded mongooses, reproductive males were found to have reduced oxidative stress in their ejaculates, supporting the oxidative shielding hypothesis, which suggests that males may play a role in minimizing oxidative stress in their offspring.

Overall, this Special Issue highlights the complex interplay between oxidative stress, reproduction, and environmental factors, with emphasis on the benefits of antioxidant supplementation, the resilience of fertility despite oxidative challenges, and the need for further ecological research to understand the reproductive costs and adaptive mechanisms associated with oxidative stress.
